# Dysfunctional intercellular communication and metabolic signaling pathways in thin endometrium

**DOI:** 10.3389/fphys.2022.1050690

**Published:** 2022-11-24

**Authors:** Liang Xu, Yingying Fan, Jianjun Wang, Rui Shi

**Affiliations:** ^1^ Research Center for Translational Medicine, Shanghai East Hospital, Tongji University School of Medicine, Shanghai, China; ^2^ Department of Obstetrics and Gynecology, Shanghai East Hospital, Tongji University School of Medicine, Shanghai, China

**Keywords:** cell diversity, thin endometrium, cell-cell communication, metabolic signaling, single-cell sequencing

## Abstract

**Background:** The endometrial thickness is a key factor for successful implantation. Thin endometrium is associated with lower implantation rate and pregnancy rate. Lacking of a better understanding for the cellular and molecular mechanisms of thin endometrium, managing patients with thin endometrium still represents a major challenge for clinicians.

**Methods:** In this study, we combined four single-cell RNA sequencing (scRNA-seq) and one bulk sequencing (bulk-seq) data for thin endometrium to perform an integrated analysis for endometrial cells in proliferating phase. Cell proportion and differentially expressed genes (DEGs) were analyzed to determine the disease-specific cell type and signaling pathways. The cell-cell communication among cell types were inferred by “CellChat” to illustrate the differential intercellular communication under normal and thin endometrium conditions. GSEA and GSVA were applied to identify dysfunctional signals and metabolic pathways before and after thin endometrium.

**Results:** Integration of scRNA-seq identified eight cell types. The proportion of stromal cells showed a significant difference between normal and thin endometrial tissue. The DEGs in diverse cell types revealed enriched pathways in a cell-specific manner. Aberrant cell-cell signaling transduction was found in almost all cell types, especially in immune cells and epithelial cells. Furthermore, dysfunctional metabolic signaling pathways were induced in a cell-type dependent way. The down-regulation of carbohydrate metabolism and nucleotide metabolism was observed and the energy metabolism switch was indicated.

**Conclusion:** Conclusively, we discover dysfunctional signals and metabolic pathways in thin endometrium, providing insight into mechanisms and therapeutic strategies for the atrophic endometrium.

## Introduction

Assisted reproductive technology is useful for the infertile women. Despite advances in assisted reproduction, the rates of successful embryo implantation are still low. Endometrial receptivity is a critical feature for pregnancy achievement. Endometrial thickness is widely used to assess the state of endometrial receptivity. While there is no consensus on a cut-off value for “thin endometrium,” thin endometrium is reported as <7 mm on the day of ovulation or on the day of human chorionic gonadotrophin (HCG) injection in fresh *in vitro* fertilization (IVF) cycles, or when using progesterone in frozen-thawed embryo transfer cycles (Liu et al., 2019). The prevalence of thin endometrium is 24–85 cases per 1,000 individuals (Kasius et al., 2014; Ribeiro et al., 2018). Thin endometrium not only implicates lower pregnancy rate, but also seems to be associated with adverse perinatal outcomes.

The most common causes of thin endometrium are inflammation and iatrogenic damage. The inappropriate endometrium repair after curettage results in disrupted blood vessel distribution and sparse glands. What’s more, thin endometrium can also be genetic and idiopathic. Currently, the detailed mechanism of thin endometrium remains unclear, and the treatments of thin endometrium are limited and controversial. Therefore, exploring the cellular and molecular mechanisms of thin endometrium, unraveling the function of different cell types, and determining the dysfunctional signaling pathways are essential for recognition of etiology and development of effective therapies.

Single-cell RNA sequencing (scRNA-seq) is a revolutionary tool that allows scientists to analyze the cell composition in tissues, identify the transcriptional states of multiple cell types, and depict the sophisticated alterations between normal and disease conditions. As a high-throughput sequencing method, scRNA-seq can provide massive information related to cell diversity and transcriptional signatures. In the present study, we integrated four scRNA-seq projects and one bulk-seq project for thin endometrium to explore the cell heterogeneity in the endometrial tissue, infer the intercellular communication between normal and diseased conditions, and reveal the metabolic alteration that contributed to thin endometrium pathogenesis.

## Materials and methods

### scRNA-seq data acquisition

We included four projects for the scRNA-seq data analysis: E-MTAB-10287 (Garcia-Alonso et al., 2021) and GSE111976 (Wang et al., 2020) contained the normal endometrial samples, and PRJNA784021 (Zhang et al., 2022) and PRJNA730360 (Lv et al., 2022) comprised of normal and thin endometrial samples. The processed matrices of E-MTAB-10287 was accessed and downloaded from human cell atlas (www.reproductivecellatlas.org). The counts matrices of normal samples of GSE111976 were downloaded from the GEO database, which contained the scRNA-seq data of two healthy donors. The raw fastq files of PRJNA784021 and PRJNA730360 were downloaded from European Nucleotide Archive (www.ebi.ac.uk/ena/). To unify the endometrial samples in the same menstrual cycle, we only included the samples in the proliferating phase from each project. As a result, we obtained nine normal samples and seven thin endometrium samples from four projects. The project information was summarized in Supplementary Table S1.

### scRNA-seq data processing

The 10x scRNA-seq data of PRJNA784021 and PRJNA730360 were processed according to 10x genomics workflow. Briefly, reads were processed using cellranger 6.0.1 pipeline with the default parameters. The Fastq files were aligned to the human reference genome (refdata-gex-GRCh38-2020-A) using the STAR algorithm. The output files were then imported into the Seurat (4.0.5) R toolkit (Stuart et al., 2019; Mimitou et al., 2021) to construct seurat objects. The seurat objects of E-MTAB-10287 and GSE111976 were constructed from matrix with the min. cells = 3, and min. features = 200. Due to the different sequencing depth, we adopted a dynamic filtration criterion by detecting outliers based on the median absolute deviation (MAD). This method has been applied by other groups, and works well in the quality control process of the single-cell data (Nguyen et al., 2018; ATL et al., 2019; Lin et al., 2021). We performed the low-quality cell filtration based on the following criteria: 1) The cells with the number of features and the number of counts not in the range of median ± 3 × MAD were removed; 2) The cells with the percentage of mitochondrial and ribosomal genes more than median + 3 × MAD were removed; 3) The cells expressing hemoglobin genes were removed, and 4) The doublets identified by DoubletFinder (version 1.0.0) (McGinnis et al., 2019) were removed. After filtration, 66,711 single cells were remained for further analysis.

### Analysis of scRNA-seq data

The R package Harmony (0.1.0) (Korsunsky et al., 2019) was used to integrate all samples. All the seurat objects from each project were SCTransformed and merged into an integrated dataset. Then the RunPCA and RunHarmony commands were used to generate harmonized dimension reduction components using sample ID and disease conditions as the grouping variable. We performed clustering using the FindNeighbors and FindClusters commands with 30 principle components at the resolutions of 0.1. The R package singleR (1.10.0) (Tucker et al., 2020) was used to annotate cell types automatically with the in-house built references, and cell types were confirmed by typical marker genes.

Detection of differentially expressed genes (DEGs) was performed using the “findMarkers” function in the Seurat R package with the “Wilcoxon” significance test for each cluster or for specific cell type between disease conditions. For the DEGs between normal and thin endometrium conditions, the threshhold was set as *p* < 0.01, pct.1 and pct.2 > 0.2, and |log2FC| > 1.

### Identification of DEGs in the bulk-seq data of thin endometrium

The raw data of thin endometrium bulk-seq data were downloaded from European Nucleotide Archive (PRJNA673823). The reads from normal and thin endometrium samples were processed to remove adapters by fastp (Chen et al., 2018), map against the GRCh38.p13.genome by hisat2 (Kim et al., 2019). And count genes by featureCounts (Liao et al., 2014). The edgeR package (Love et al., 2014) was used to determine the DEGs against the control condition.

### Functional enrichment analysis

The R package clusterProfiler (4.0.5) (Yu et al., 2012; Wu et al., 2021) was used to investigate the function of DEGs. Biological significance of DEGs was inferred by GO term enrichment analysis. All the sub-ontology biological process, cellular component, and molecular function were demonstrated. The critical pathways closely related to thin endometrium was identified by KEGG pathway enrichment analysis. A *p* < 0.05 was considered significant. To visualize the KEGG annotation across clusters between normal and thin endometrium conditions, we adopted “compareCluster” with the function “enrichKEGG.”

### Gene set enrichment analysis for thin endometrium bulk-seq data

We applied GSEA analysis by the clusterProfiler R package (4.0.5) with the function “GSEA.” The pathways were selected from the MSigDB/GSEA resource c2.cp.reactome.v7.4.entrez.gmt (https://www.gsea-msigdb.org/gsea/msigdb/). The whole genes in the bulk-seq data were ordered by log2FC, and subjected to GSEA analysis. The *p*-value cut-off for the enriched pathways was set as 0.05. We randomly labeled five genes in each pathway.

### Analysis for intercellular communication

The cell-cell communication was inferred by the R package CellChat (1.1.3) (Jin et al., 2021) with all the built-in database including “secreted signaling,” “ECM-receptor,” and “Cell-Cell Contact.” Using the normalized count and clustering information as the input, CellChat can compare intercellular communications for cell populations based on the known structural composition of ligand-receptor interactions.

### Single sample gene set enrichment analysis

The R package GSVA (1.40.1) was used for differential metabolic pathways with the method of ssGSEA. The gene sets were obtained from published study containing metabolic gene sets from KEGG and REACTOME database (Wu et al., 2022). The average expression of the integrated scRNA-seq data in each cluster was computed to compare the enrichment levels of metabolic signatures among clusters. For the enrichment of the metabolic signatures in each cluster under normal and thin endometrium conditions, the score of metabolic pathways was computed for each cell, and grouped by clusters. The R package “limma” (Ritchie et al., 2015) was used for determining the significance between normal and thin endometrium conditions in each cluster.

### Statistical analysis

The statistical analysis was performed by GraphPad Prism 8 (GraphPad Software, San Diego, CA, United States) For normally distributed data, the unpaired *t*-test and ANOVA analysis were performed. For non-normal data, we used the non parametric test Mann-Whitney test. *p* < 0.05 was considered as significant.

## Results

### Combination of scRNA-seq data from normal and thin endometrial samples in proliferating phase

**FIGURE 1:**
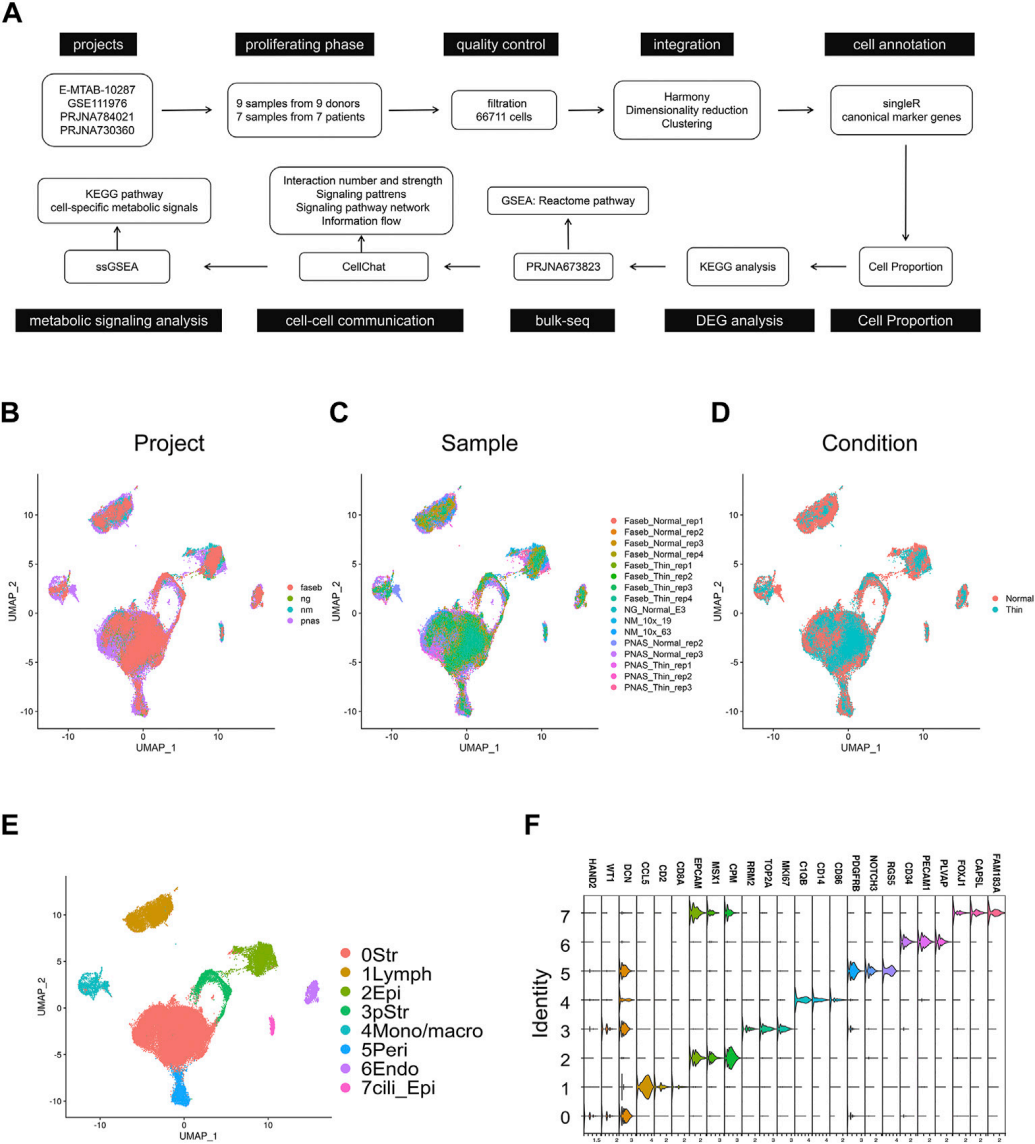
Integration of scRNA-seq projects for human normal endometrial tissue and thin endometrial tissue. **(A)** Workflow of scRNA-seq and bulk-seq data processing and analyzing for thin endometrial tissue. UMAP visualization showing the integrated effects of 66,711 cells in projects **(B)**, samples **(C)**, and conditions. **(D)**. **(E)** UMAP of cells demonstrating 8 major cell types. Str, stromal cell; Lymph, lymphoid cells; Epi, epithelial cells; pStr, proliferating stromal cells; Mono/macro, mono/macrophages; Peri, pericytes; Endo, endothelial cells; and Cili_Epi, ciliated epithelial cells. **(F)** Violin plot showing the expression level of the marker genes in each cell type.

We obtained processed matrices and raw sequencing data of four studies: E-MTAB-10287 (ng), GSE111976 (nm), PRJNA784021 (faseb), and PRJNA730360 (pnas) (Figure 1A). To compare the endometrial cells across datasets, we selected the cell data in the proliferating phase from each project. The detailed information for each dataset was summarized in Supplementary Table S1. After stringent filtration (Supplementary Figure S1A), 66,711 cells were gathered and combined by Harmony method (Supplementary Figure S1B). The UMAP low dimensional space showed the similar distribution in projects, samples and conditions (Figures 1B–D; Supplementary Figure S2). We used the unsupervised graph-based clustering to find eight clusters of endometrial cells (Figure 1E) and defined the main cell types by SingleR (Tucker et al., 2020). The cell identities were further validated by typical cell markers (Figure 1F; Supplementary Table S2). As a result, the final integrated dataset was identified as stromal cells, lymphoid cells, epithelial cells, proliferating stromal cells, mono/macrophages, pericytes, endothelial cells, and ciliated epithelial cells).

### Cell population alteration in thin endometrium

**FIGURE 2:**
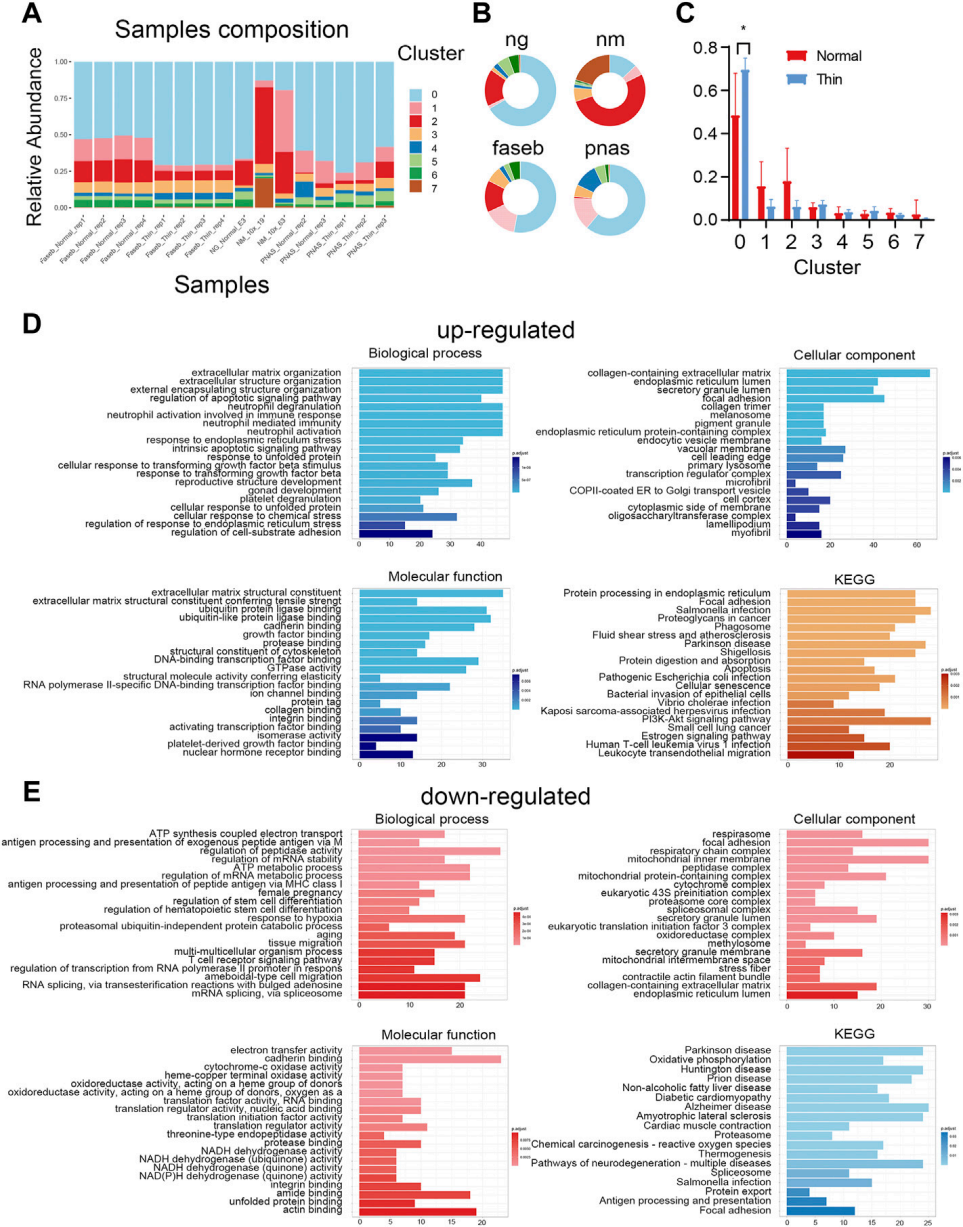
Differential cell population in thin endometrial tissue. **(A)** The cell type composition for each sample. **(B)** The sample profiles in individual projects. **(C)** The proportions of cell types in normal and thin endometrial tissues. *p < 0.05. The functional annotation for the upregulated **(D)** and downregulated **(E)** DEGs in stromal cells.

The cell diversity plays the functional role in the endometrial tissue, but thin endometrium exhibited comparable cell types to that of normal endometrial samples, so we next explored the alteration of cell proportion in thin endometrium. As shown in Figures 2A,B, the variation of cell compositions in samples and projects were relatively large. We only found the significant difference in stromal cells between normal and thin endometrial tissues (Figure 2C). The stromal cells make up the largest proportion of the endometrium and control tissue proliferation, remodeling, and breakdown during the menstrual cycle (Queckbörner et al., 2020). We further examined the differential pathways by up- and downregulated genes in stromal cells under normal and thin endometrium conditions. The GO and KEGG analysis revealed the cell-type specific regulation for the up- and downregulated genes in stromal cells. For instance, the increased gene expression in stromal cells was associated with the biological functions in extracellular matrix organization, response to endoplasmic reticulum stress, and neutrophil activation, and with the pathways in apoptosis, cellular senescence, and estrogen signaling pathway (Figure 2D). By contrast, the decreased genes were involved in the function of regulation of mRNA metabolic process, female pregnancy, and regulation of stem cell differentiation. These genes were linked to oxidative phosphorylation, protein export, and antigen processing and presentation (Figure 2E). These results imply that the decreased cell proportion of stromal cell are important in the progress of thin endometrium.

### Cell-specific alterations of signaling pathways in thin endometrium

**FIGURE 3:**
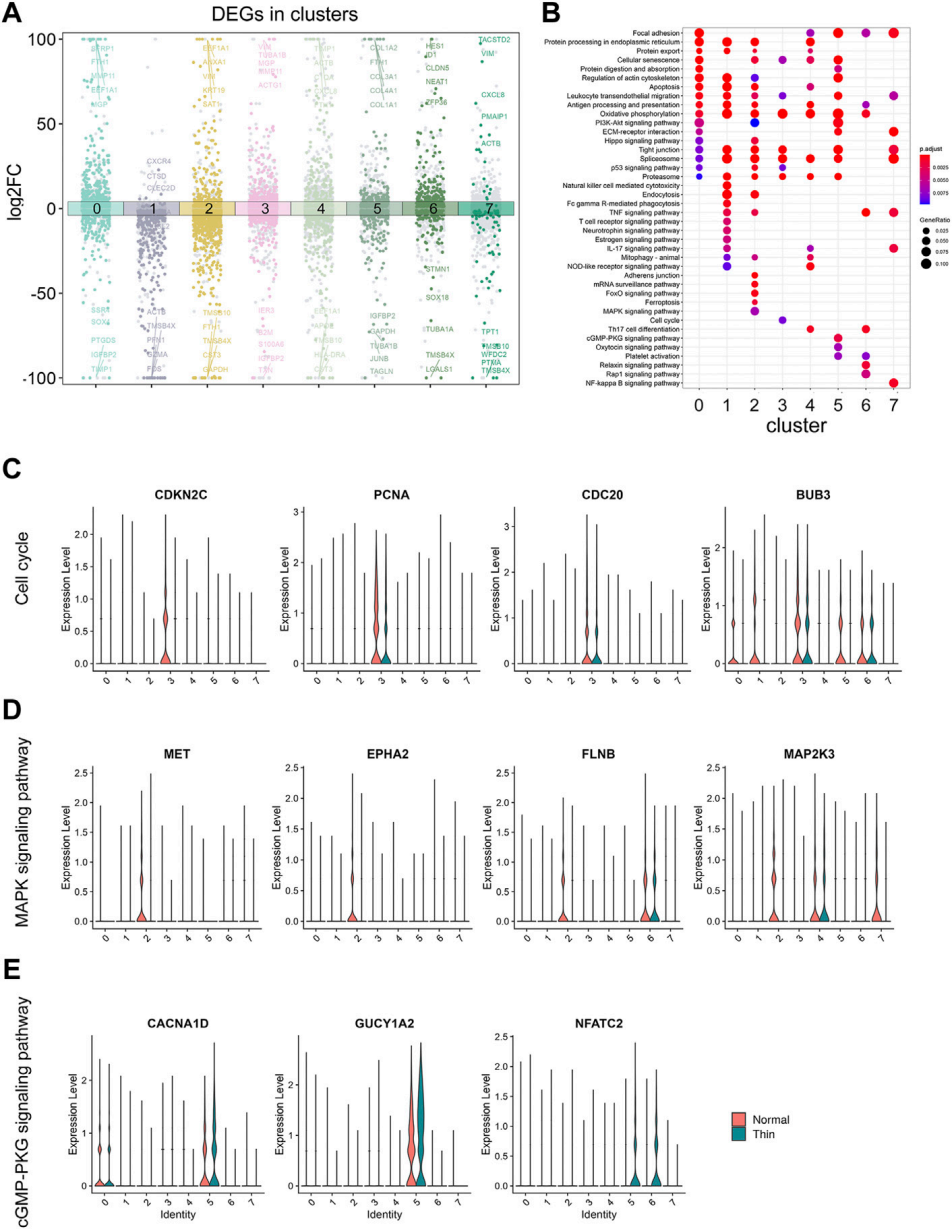
The analysis for DEGs in clusters and under thin endometrium condition. **(A)** Dot plot showing the up- and downregulated genes across all eight clusters in thin endometrial tissue. The DEGs were colored by clusters and labeled by the top5 up- and downregulated DEGs in each cluster. **(B)** KEGG enrichment of upregulated and downregulated DEGs across clusters. Violin plot showing the expression level of genes in enriched KEGG pathways: cell cycle **(C)**, MAPK signaling pathway **(D)**, and cGMP- PKG signaling pathway **(E)**.

We next analyzed the gene expression under normal and thin endometrium conditions to reveal the function of DEGs in each cluster. A total of 4,306 DEGs were found (Supplementary Table S3), and illustrated with the top5 DEGs (upregulated and downregulated) labeled in each cluster (Figure 3A). We looked for over- or under- represented KEGG gene sets in our DEG list. Our results revealed the common and unique signaling pathways across clusters (Figure 3B). The oxidative phosphorylation was involved in almost all clusters. Generally, most genes in the oxidative phosphorylation pathway were downregulated under the thin endometrial condition (Supplementary Figure S3), suggesting an impaired energy metabolism in thin endometrium. The tight junction and splicesome were also demonstrated in almost all clusters. In contrast to the universal pathways, the natural killer cell mediated cytotoxicity, T cell receptor signaling pathway, and Fc gamma R-mediated cytotoxicity occurred in lymphoid cells; the Ferroptosis, FoxO, and MAPK signaling pathway played specially a role in epithelial cells; and Relaxin and Rap1 signaling pathways were only in endothelial cells. These altered signaling pathways indicate cell-type specific response in thin endometrium.

We further examined the expression of the signaling genes at the single cell level. As shown in Figure 3C, the cell cycle was significant in proliferating stromal cells, and the pathway genes, CDKN2C, PCNA, CDC20, and BUB3 were significantly decreased in proliferating stromal cells. Similarly, the genes, MET, EPHA2, FLNB, and MAP2K3 were downregulated in epithelial cells, and the genes, CACNA1D, GUCY1A2, and NFATC2 were elevated in pericytes, consistent with the findings in MAPK and cGMP-PKG signaling pathways among clusters (Figures 3D, E). These results demonstrated the disease-induced and cell-specific pathway alterations in thin endometrium.

### Bulk-seq revealed the differential pathways in thin endometrium

**FIGURE 4:**
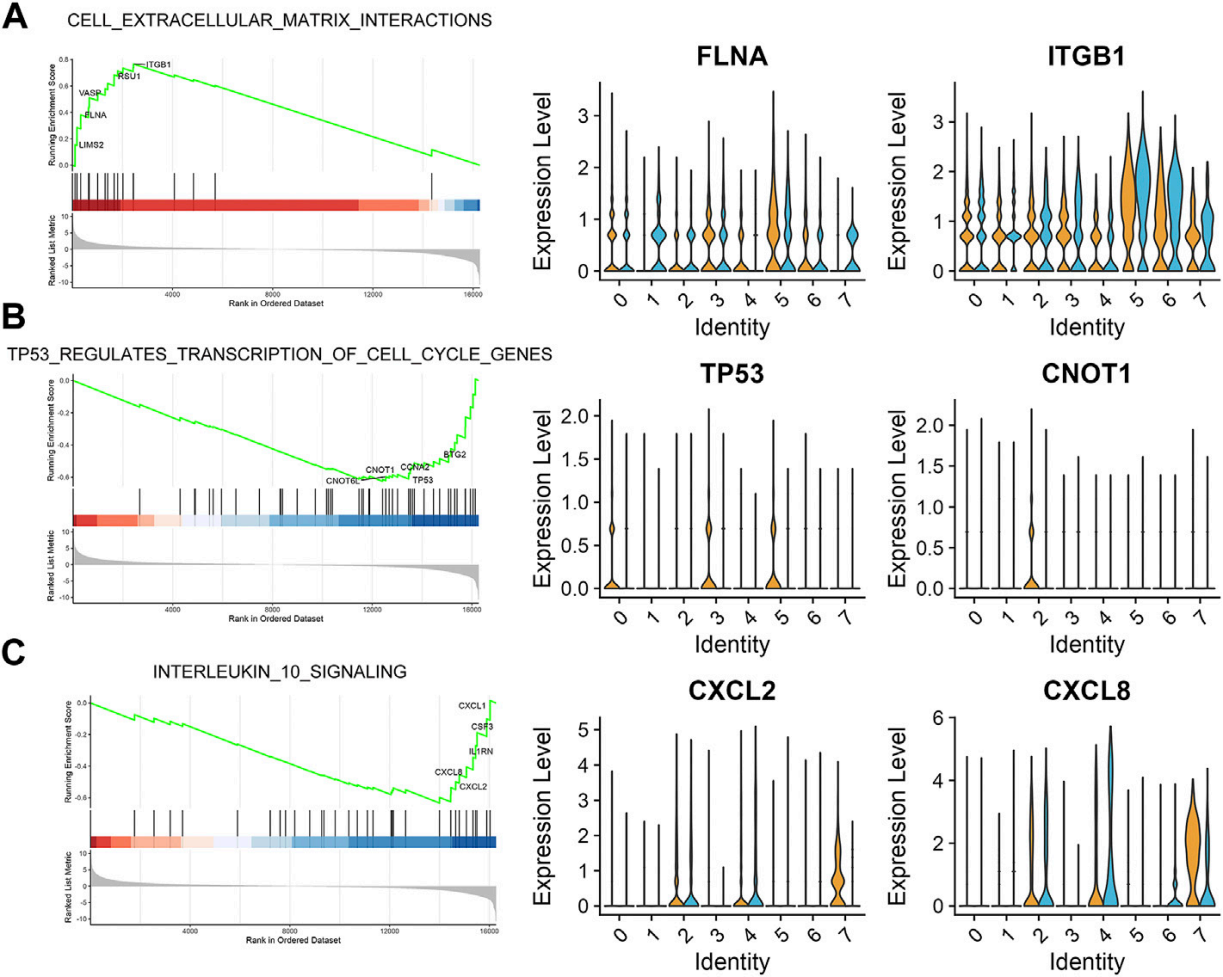
Bulk-seq revealed the enriched pathways in thin endometrium. GSEA analysis showing the pathways enriched in the top **(A)** and bottom **(B,C)** of the ranked list, with the corresponding up- or downregulated genes. *Left*, combo chart showing the running enrichment score and ranked list metric for the ordered fold change gene list with 5 pathway genes labeled; *right*, the expression level of the representative genes in each cluster.

In addition to scRNA-seq data, we introduced a bulk-seq (GSE160633) data to uncover common signals affected in thin endometrium. We used edgeR to identify the up- and downregulated DEGs in the data (Supplementary Table S4), and performed GSEA analysis to determine the putative functional alteration in thin endometrium. Our data revealed up-regulation of pathway associated with cell extrcellular matrix interaction and down-regulation of TP53 regulation transcription of cell cycle genes and interleukin 10 signals (Figures 4A–C). We further checked the expression of the pathway-associated genes at the single cell level. In the cell extracellular matrix interactions, the expression of FLNA and ITGB1 was significantly elevated in lymphoid cells, pericytes, endothelial cells, and ciliated epithelial cells. By contrast, the decreased expression of TP53, and CNOT1 in TP53 signals occurred in stromal cells, epithelial cells and proliferating stromal cells. Interestingly the reduction of CXCL2 and CXCL8 expression was mainly attributed to ciliated epithelial cells. These results suggest the abnormal pathways in the development of thin endometrium.

### Dysfunctional cell-cell communication in thin endometrium

**FIGURE 5:**
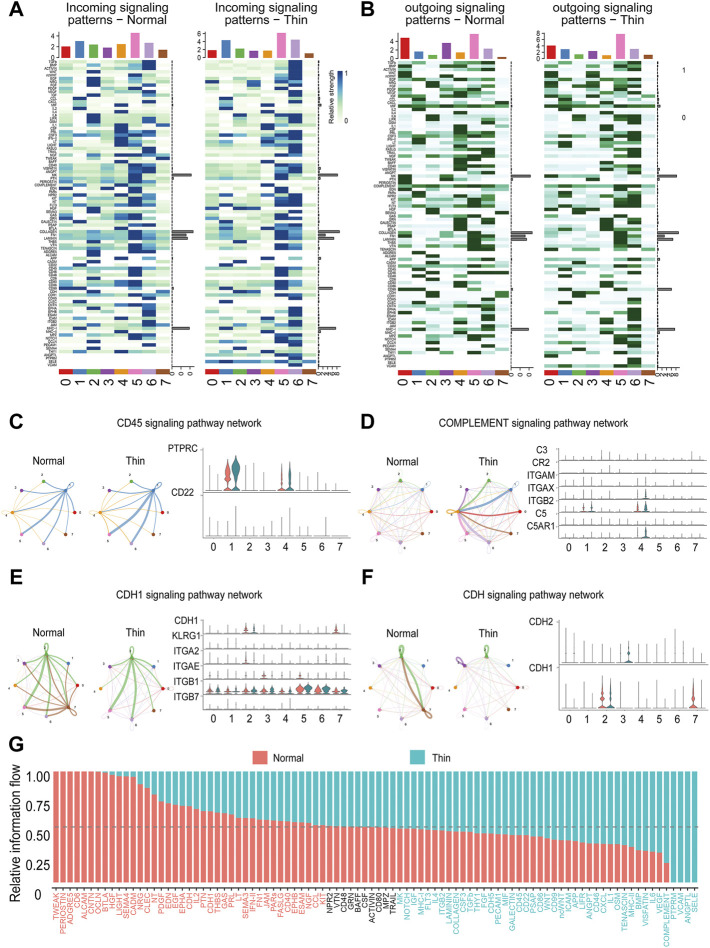
Dysfunctional cell-cell communication in thin endometrium. Heatmap depicting differential incoming **(A)** and outgoing **(B)** signaling patterns in each cluster. Representative networks for enhanced **(C,D)** and reduced **(E,F)** signaling pathways. *Left*, circle plot showing the signals in cell-cell interactions between normal and thin endometrium; *right*, the expression level of ligand-receptor genes in individual signals. **(G)** Bar graph demonstrating the relative information flow of each signaling pathway between normal and thin endometrial tissues.

Cell-cell communication is pivotal for regulating individual cell processes and intercellular relationships. We adopted “CellChat” to infer the communication network by integrating paired ligand-receptor genes. We found that while the global number of interactions was reduced, the strength of interactions was increased (Supplementary Figure S4A). Each cluster had differential signals in incoming and outgoing patterns under normal and thin endometrial conditions (Figures 5A,B). Overall, the number and strength of interactions in stromal cells, proliferating stromal cells and mono/macrophages were both decreased, and the interactive numbers and strength of lymphoid cells, and endothelial cells were both increased (Supplementary Figure S4B,C). Strikingly, the pathways involved in immune and inflammatory responses were enhanced. As shown in Figures 5C,D, the CD45 and complement signaling pathway networks were boosted, and the genes in these signaling pathways were upregulated accordingly. The signals in ciliated epithelial cells were severely impaired (Figures 5A,B). This was prominent in the CDH1 and CDH signaling pathway networks and corresponding gene expression (Figures 5E,F).

We further explored the overall information flow of each signaling pathway to dissect the alteration of signaling pathways in thin endometrium (Figure 5G). The information flow was defined by the sum of communication probability among all pairs of cell groups in the inferred network (Jin et al., 2021). The signaling pathways, including TWEAK, PERIOSTIN, ADGRE5, CD6, ALCAM, and CNTN, which occurred in normal endometrial tissue, were shut off in thin endometrium, whereas PTPRM, VCAM, ANGPTL, and SELE, which were not activated in normal endometrial tissue, were turn on in thin endometrium. A few signals such as NPR2, VTN, CD48, GRN, BAFF, CSF, ACTIVIN, CD80, MPZ, and TRAIL were sustainable activated. And HGF, LIGHT, SEMA4, CADM, BMP, VISFATIN, IL6, and VEGF were dynamically changed. These results manifest that the cell-cell interaction network was disturbed in thin endometrium.

### Dysfunction of metabolic pathways in thin endometrium

**FIGURE 6:**
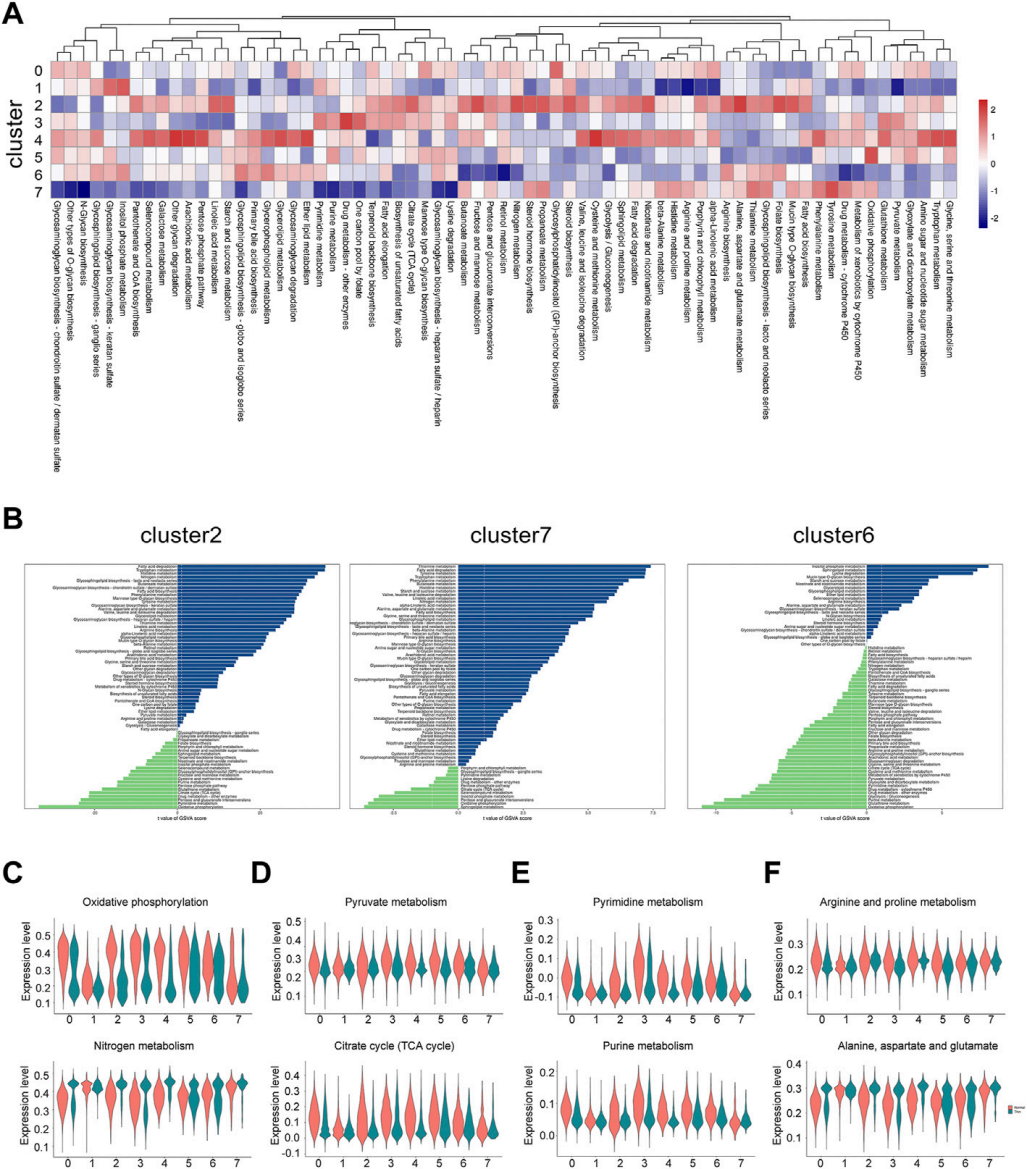
The metabolic alteration in individual cell types under thin endometrium condition. **(A)** Heatmap showing the differential metabolic pathways in each cell type. **(B)** Bar plot showing the dramatically changed metabolic pathways with the metabolic gene sets from the KEGG database in epithelial cells, ciliated epithelial cells, and endothelial cells. Violin plot showing the representative metabolic pathways in energy metabolism **(C)**, carbohydrate metabolism **(D)**, nucleotide metabolism **(E)**, and amino acid metabolism **(F)**.

Periodic regulation of cell metabolism are essential for the maintenance of normal uterine function and fertility. It may also contribute to the development of endometrial disorders. Evidence has showed that a few of enzymes and their substrates can be detected in endometrial tissues (Gibson et al., 2020). Therefore, we attempted to find the metabolic abnormality in the thin endometrial tissue. We investigated the metabolic state of each cluster by calculating the score of metabolism-associated pathways in each cluster. Generally, epithelial cells are the most dynamic cell type, and on the contrary, the ciliated epithelial cells was the most inert cell type (Figure 6A). We further explored the differential metabolic pathways in all clusters between normal and thin endometrial tissue (Supplementary Table S5). In epithelial cells, the lipid metabolism, including fatty acid degradation, fatty acid biosynthesis and glycerolipid metabolism was increased, and purine and pyrimidine metabolism were reduced. In ciliated epithelial cells, while a series of metabolic pathways including thiamine metabolism, tyrosine and tryptophan metabolism were elevated, only a few pathways such as sphingolipid metabolism and pentose and glucuronate interconversions were declined. Astonishingly, only in endothelial cells, most of the metabolic pathways were reduced (Figure 6B).

We next examined the state of metabolic pathways in each cluster in energy metabolism, carbohydrate metabolism, nucleotide metabolism, and amino acid metabolism. Some interesting results were found. The increased nitrogen metabolism was paralleled with the decreased oxidative phosphorylation (Figure 6C). The decline of pyruvate metabolism and citrate cycle were most prominent in stromal cells, mono/macrophages and pericytes (Figure 6D). The metabolic pattern of nucleotide metabolism was similar to that of carbohydrate metabolism (Figure 6E). The alteration of amino acid metabolism depended on the type of amino acids. As shown in Figure 6F, while the arginine and proline metabolism was decreased, the alanine, aspartate and glutamate metabolism was increased in the thin endometrial tissue. These data indicate the dramatic alteration of metabolic signaling in thin endometrium.

## Discussion

Endometrial thickness increases during the proliferating phase. The endometrium undergoes more than simple estrogen responsive growth in this period (Queckbörner et al., 2020). In this study, we used the high-throughput transcriptome sequencing data to uncover the dysfunctional signaling pathways and abnormal metabolic states between normal and thin endometrium. We collected over 60,000 single cells from 16 samples in the proliferating phase. After stringent quality control, all cells were identified as eight major cell types. Although disease-specific cell population was not observed, the cell proportion of stromal cells was significantly elevated in thin endometrial tissue. The stromal cells comprise the largest proportion of the endometrium. The increased ratio of stromal cells may indicate the adaptive response for thin endometrium. The upregulated gene expression was associated with extracellular matrix production, and neutrophil activation, and the downregulated gene expression was linked to aging and energy metabolism.

In this study, we explored the cell-cell communication network in thin endometrium with “CellChat.” As expected, the number and strength of interactions in stromal cells and proliferating stromal cells were attenuated. Notably, macrophages also exhibited declined interaction numbers and strength. Macrophages comprise 1%–2% of endometrial cells in the proliferating phase. Their numbers increased during menses (Brown et al., 2022). Macrophages are key effector cells in clearing cell debris and apoptotic cells during endometrial shedding. The impairment of cell talk in macrophages may hinder endometrial regeneration, and influence innate and humoural immunity. We also observed the severe destruction of cell-cell communication in ciliated epithelial cells. Ciliated epithelial cells are the epithelial cells with numerous motile cilia. The proportion of these cells is dynamically regulated during the menstrual cycle. Their main function is to actively carry the mucus along the mucous membrane. The damage of cell interactions for ciliated epithelial cells may compromise the function of endometrial protection.

By contrast, the signaling pathways related to lymphoid cells were prominently enhanced. The ligand-receptor pair genes in CD45 and complement signals were upregulated. A conspicuous number of immune cells are located in the human endometrium. Mainly they are NK cells, which phenotype are distinctive from peripheral cytotoxic NK cells and macrophages. In addition, a few B cells and CD8^+^ T cells are aggregated in the endometrial tissue. Evidence has shown that endometrial NK cells have a dedicated tissue-specific phenotype (Feyaerts et al., 2018), in the proliferating phase, only a few NK cells exist in the endometrial tissue, and they continue to increase until menstruation (Agostinis et al., 2019; Wang et al., 2021). The mis-activation of immune cells and disorder of cell dialogue to other cell types may contribute to the pathology to thin endometrium.

Metabolic heterogeneities actively participate to therapeutic failure (Kim and DeBerardinis, 2019; Zeisel, 2020), so we attempted to uncover the metabolic complexity and flexibility in thin endometrium. In the endometrial tissue, various cell types exhibit differential metabolic states (Figure 6A). Intriguingly, the epithelial cell and the ciliated epithelial cell showed completely different metabolic states. The epithelial cell maintained a high level of metabolism, whereas the ciliated epithelial cell remained inert. The similar pattern was found on lymphoid cells and macrophages. While lymphoid cells kept a low level of metabolic state, macrophages were active in most of observed metabolic pathways. Strikingly, the proliferating stromal cells were dynamic in nucleotide metabolism, one carbon metabolism, TCA cycle, and oxidative phosphorylation, representing an actively proliferating state.

Endometrial cells experienced tremendous changed in thin endometrium. The energy metabolism was switched from oxidative phosphorylation to nitrogen metabolism. The carbohydrate metabolism, and nucleotide metabolism were generally downregulated. The metabolism for fat and amino acid was cell-type dependent and pathway dependent. For example, Stromal cells and macrophages were active in most of lipid metabolism such as fatty acid biosynthesis, steroid hormone biosynthesis, and linoleic acid metabolism (Supplementary Figure S5). These metabolic processes may be potential targets to regulate disease progression.

The insufficient cell proliferation and dysfunctional cells are the distinguished features for thin endometrium. The specific gene signatures found in this study can be applied to the prognosis of thin endometrium, especially for the patients with the threshold thickness of endometrium. For example, given the fact that the proportion of stromal cells is increased and the collagen is overloaded, the genes COL1A1, COL3A1, and COL5A2, can be potential biomarkers for evaluation of severity of thin endometrium. Owing to the activation of immune system and enhancement of lymphoid cell-associated signaling pathways, the expression of CXCR4, and CTSD can be examined for the risk of thin endometrium. Moreover, increased cellular senescence and collagen overdeposition, and NK cell overactivation revealed from this study may give clues for treatment of thin endometrium targeting delaying cell ageing, clearing matrix collagen and inhibiting immune over-excition.

Our study still has several limitations. A potential limitation is the small sample size in one phase of menstrual cycle. More samples at various phase of menstrual cycle will provide more valuable evidence and identify more special traits in thin endometrial patients. Another limitation is the verification of differentially expressed genes. While the genes in cell-cell signaling transduction and metabolic signaling pathways were examined at the transcriptional level, they have not been validated at the protein level. Further studies addressing the molecular and functional basis of thin endometrium in relevant animal models and cell experiments is necessary. Multi-omics data, including proteomics and metabolomics may add more information for understanding the mechanism of thin endometrium.

## Conclusion

Collectively, we combined the scRNA-seq and bulk-seq data, performed a comprehensive analysis for the thin endometrium. Our study dispicted the cellular diversity in endometrial tissues, and identified the dysfunction of intercellular signaling transduction and the impairment of metabolic signaling pathways in thin endometrial tissues. These pathways are potential diagnostic and therapeutic targets for thin endometrium treatment.

## Data Availability

The datasets presented in this study can be found in online repositories. The names of the repository/repositories and accession number(s) can be found in the article/Supplementary Material.
