# Supplementary figures and images for: Dysfunctional intercellular communication and metabolic signaling pathways in thin endometrium
Source: Front Physiol. 2022 Nov 24;13:1050690. doi: 10.3389/fphys.2022.1050690 (PMC9729336; doi:10.3389/fphys.2022.1050690)

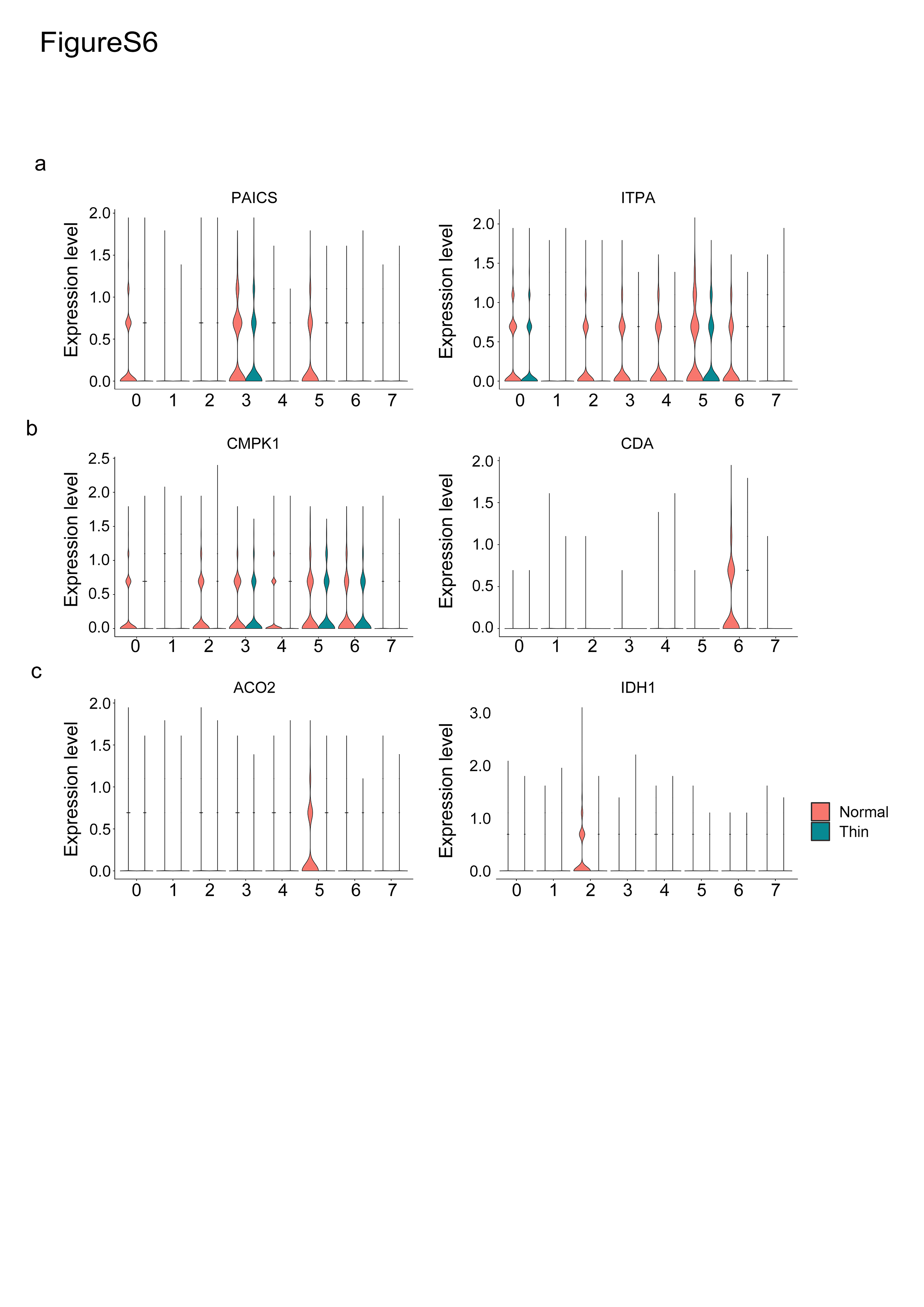

Supplement: Supplementary file 4 [file Image6.TIF]

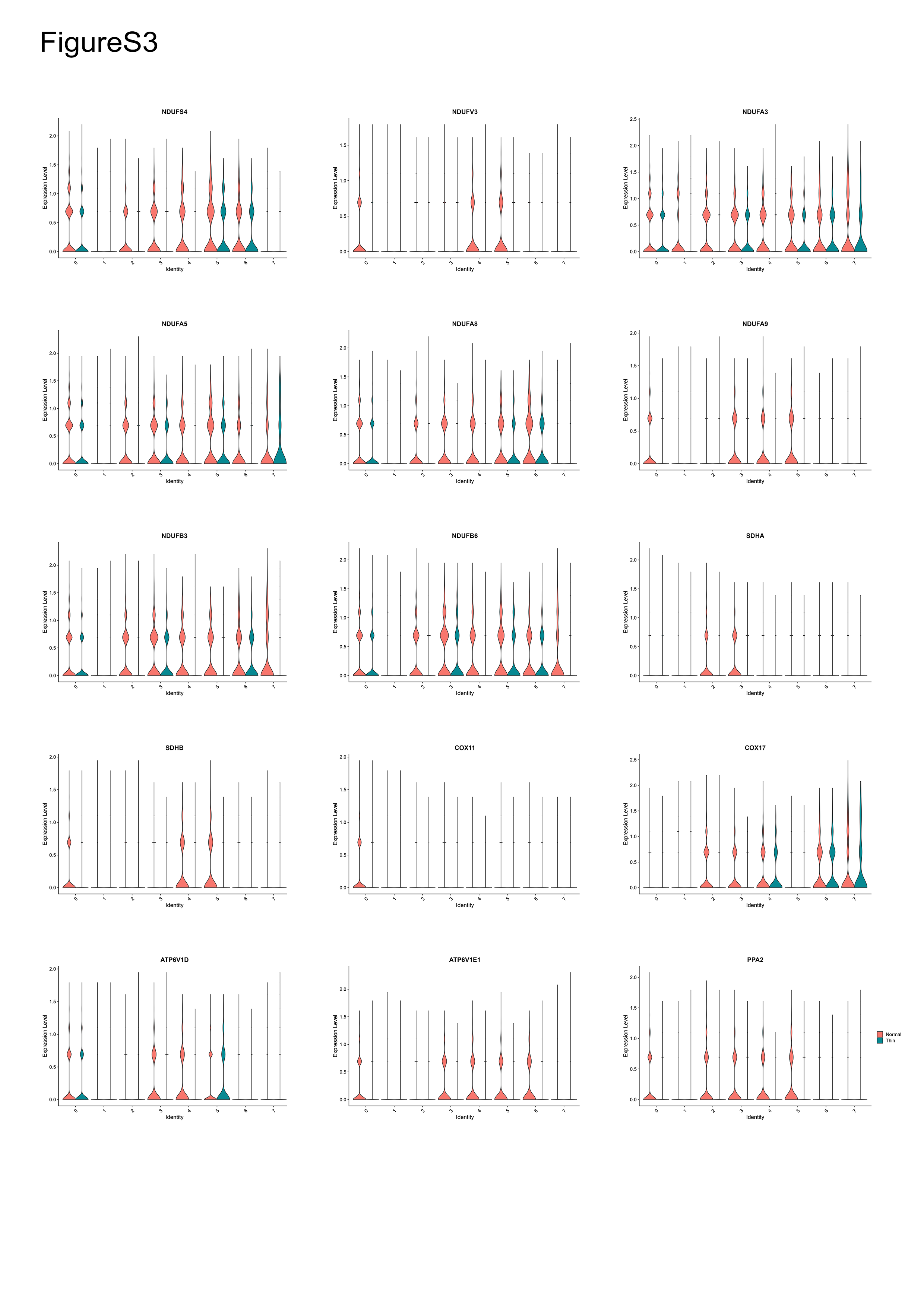

Supplement: Supplementary file 5 [file Image3.TIF]

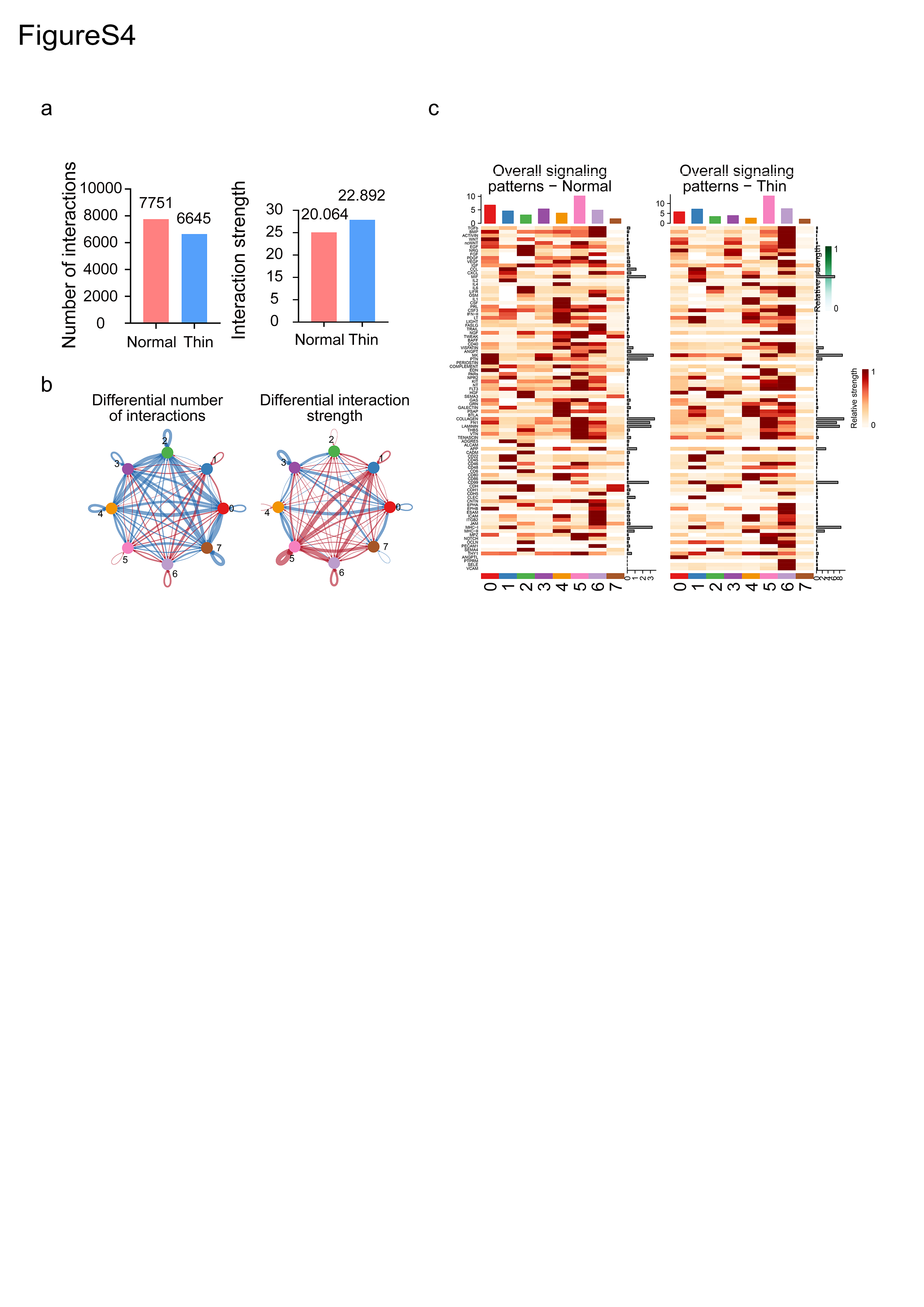

Supplement: Supplementary file 6 [file Image4.TIF]

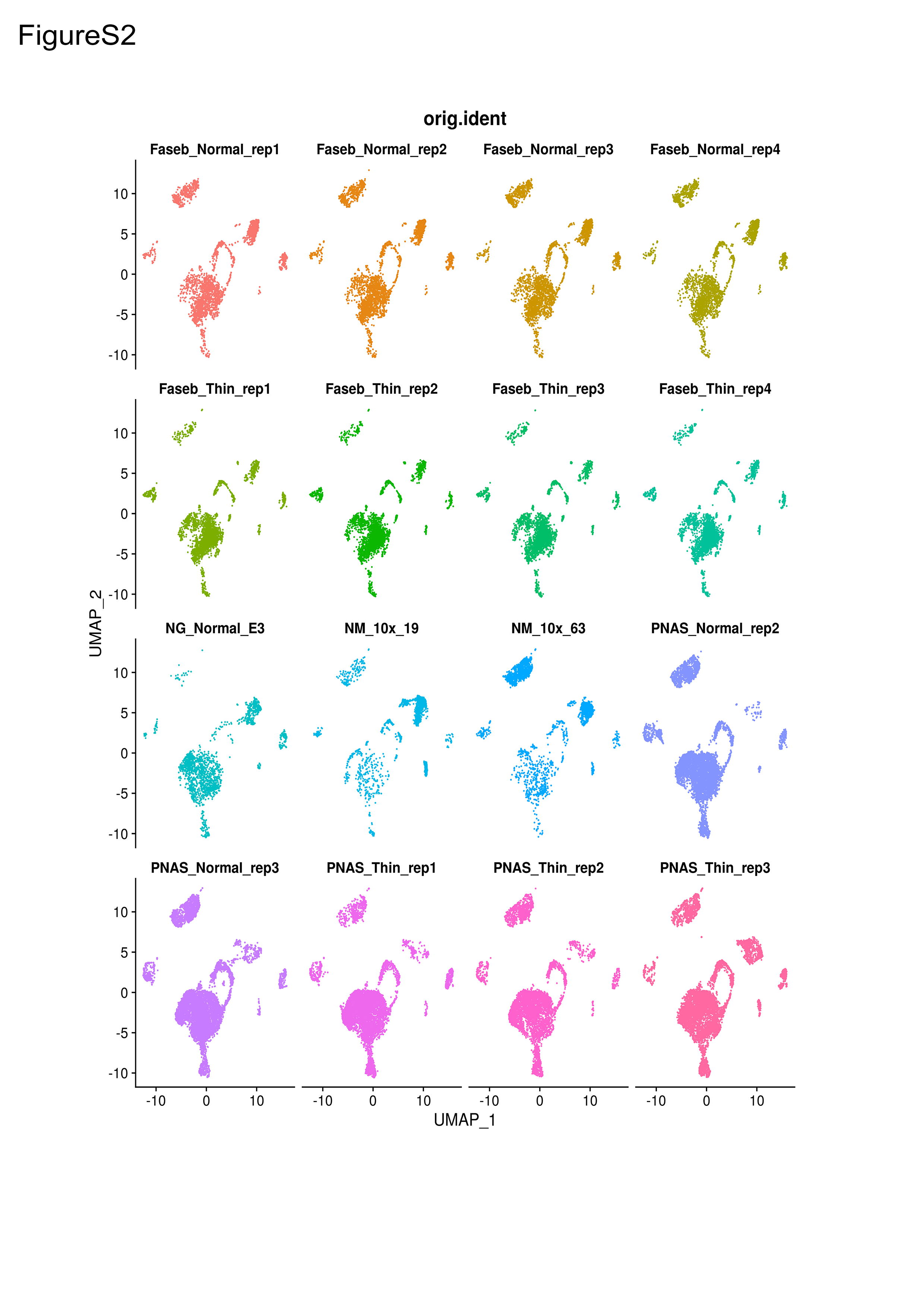

Supplement: Supplementary file 8 [file Image2.TIF]

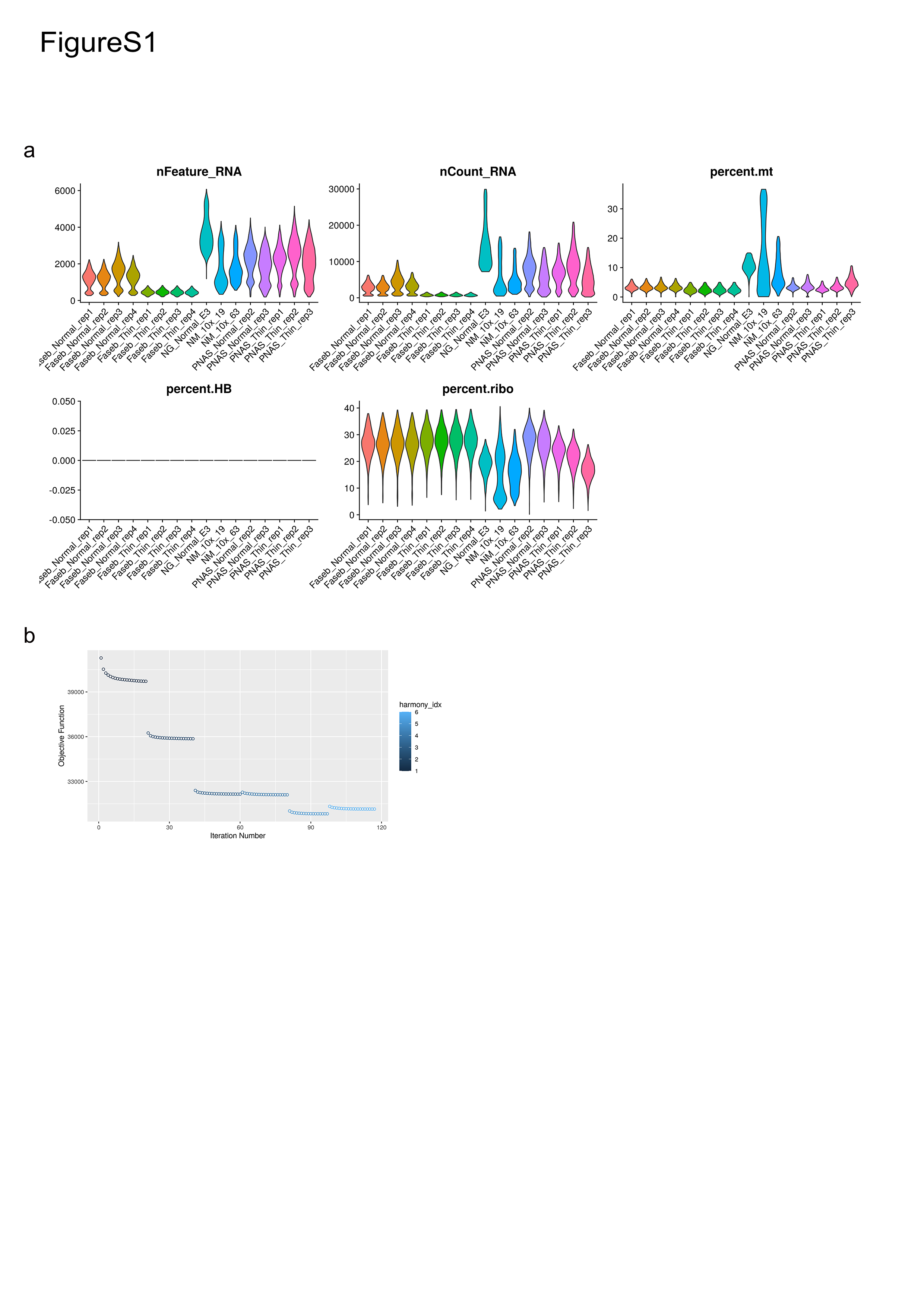

Supplement: Supplementary file 10 [file Image1.TIF]

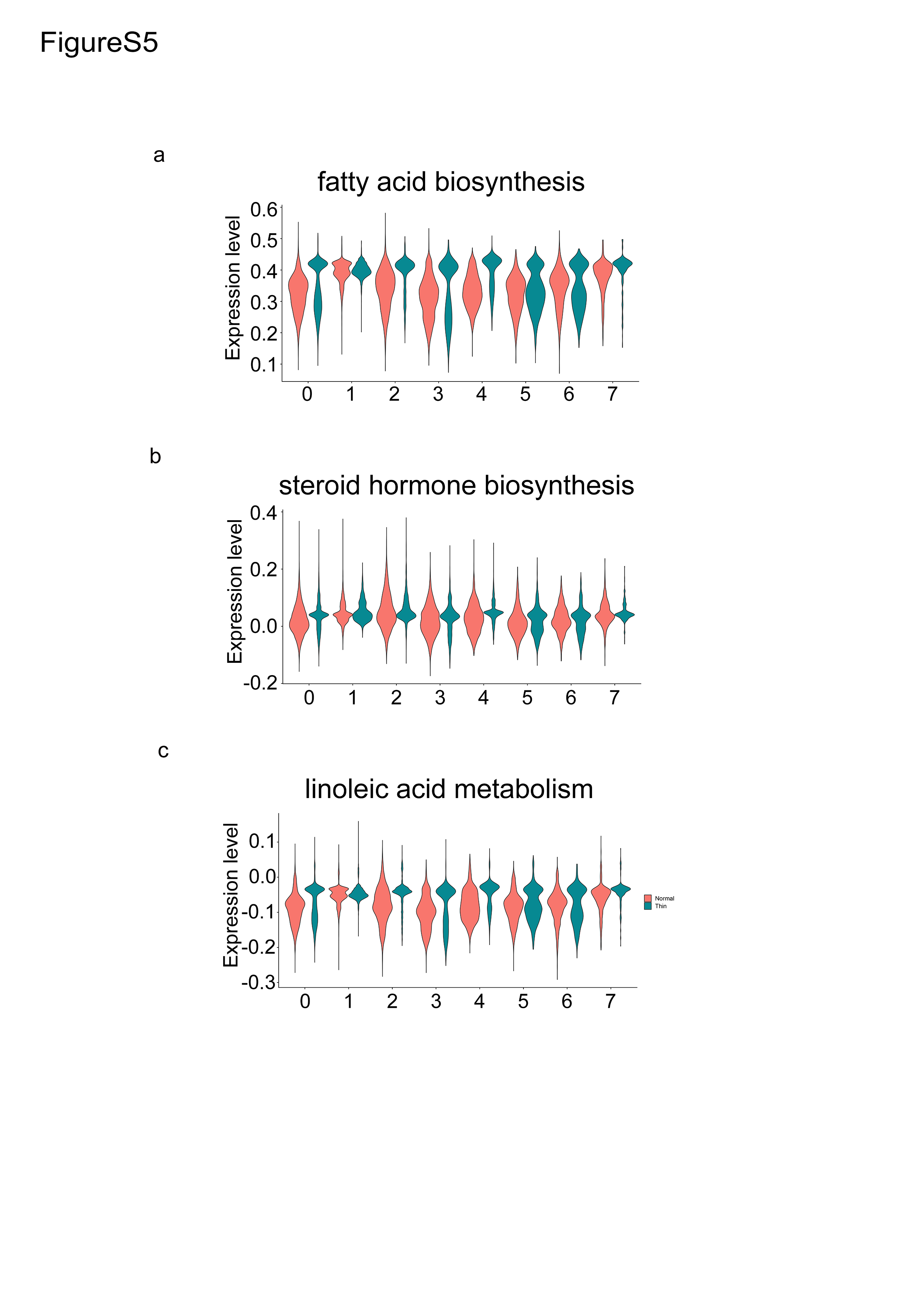

Supplement: Supplementary file 11 [file Image5.TIF]
