# Supplementary material for: Dysfunctional intercellular communication and metabolic signaling pathways in thin endometrium
Source: Front Physiol. 2022 Nov 24;13:1050690. doi: 10.3389/fphys.2022.1050690 (PMC9729336; doi:10.3389/fphys.2022.1050690)
Supplement: Supplementary file 12 [file DataSheet1.DOCX]

**Supplementary Information**

Table. S1. Information of the integrated scRNA-seq projects.

Table. S2. DEGs in individual cell types.

Table. S3. DEGs in each cell type between normal and thin endometrium.

Table. S4. DEGs between normal and thin endometrium in bulk-seq project.

Table. S5. Differentially metabolic pathways in each cluster between normal and thin endometrium.

**Supplementary Figure legends**

**Figure S1. Integration of four scRNA-seq projects in endometrial tissues.** (a) The quality control of 16 endometrial samples from 4 datasets. (b) Integration process by Harmony.

**Figure S2. Cell distribution of individual samples in the integrated data.** UMAP plot showing the cell distribution of each sample.

**Figure S3. Gene expression in oxidative phosphorylation pathway under thin endometrium condition.** Violin plot showing the expression of genes in oxidative phosphorylation pathway between normal and thin endometrium.

**Figure S4. Intercellular communication inference between normal and thin endometrium.** (a) Bar plot showing the interaction numbers and strength between normal and thin endometrium. (b) Circle plot showing the overall differential number of interactions and interaction strength. (c) Heatmap depicting the overall signaling patterns in normal and thin endometrium. .

**Figure S5. Lipid metabolism in thin endometrium.** Violin plot showing the representative fatty metabolic signals: (a) fatty acid biosynthesis, (b) steroid hormone biosynthesis, and (c) linoleic acid metabolism.

**Figure S6. Differentially gene expression in metabolic pathways under thin endometrium condition.** Violin plot showing the expression of representative genes in (a) purine metabolism, (b) pyrimidine metabolism, and (c) citrate cycle (TCA cycle).
